# Low-Molecular-Weight Heparin in Preeclampsia: Effects on Biomarkers and Prevention: A Narrative Review

**DOI:** 10.3390/biomedicines13102337

**Published:** 2025-09-24

**Authors:** Dimitris Baroutis, Konstantinos Koukoumpanis, Alexander A. Tzanis, Marianna Theodora, Konstantinos Rizogiannis, Dimitrios Bairaktaris, Efstathios Manios, Vasilios Pergialiotis, Evangelos Alexopoulos, George Daskalakis

**Affiliations:** 1 1st Department of Obstetrics & Gynecology, Alexandra Hospital, National and Kapodistrian University of Athens, 11528 Athens, Greece; 2 Department of Clinical Therapeutics, Alexandra Hospital, National and Kapodistrian University of Athens, 11528 Athens, Greece; 3 Gynäkologie und Geburtshilfe, Kantonsspital Winterthur, 8400 Winterthur, Switzerland

**Keywords:** preeclampsia, low-molecular-weight heparin, prevention, biomarkers, placenta-mediated complications, thromboprophylaxis, angiogenic factors, pregnancy complications

## Abstract

Preeclampsia affects 2–8% of pregnancies globally and remains a leading cause of maternal and perinatal morbidity, with limited preventive options beyond low-dose aspirin. Low-molecular-weight heparin (LMWH) has emerged as a promising therapeutic candidate due to its pleiotropic effects extending beyond anticoagulation, including anti-inflammatory, pro-angiogenic, and placental-protective properties. This comprehensive narrative review examines LMWH’s effects on preeclampsia-associated biomarkers and evaluates clinical evidence for its preventive efficacy. LMWH exerts multifaceted effects on disease pathophysiology, including restoration of angiogenic balance through sFlt-1 reduction and PlGF preservation, attenuation of inflammatory responses via decreased TNF-α and IL-6 production, normalization of coagulation parameters, and enhancement of trophoblast invasion and placental vascularization. Clinical trials reveal heterogeneous results, with meta-analyses suggesting significant benefit primarily in high-risk subgroups. Women with previous severe placenta-mediated complications demonstrate relative risk reductions of 40–60% for recurrent preeclampsia with LMWH prophylaxis, particularly when initiated before 16 weeks’ gestation. Combination therapy with low-dose aspirin appears to enhance protective effects. However, larger trials in unselected populations have failed to demonstrate significant benefit, highlighting the importance of appropriate patient selection. Current international guidelines reflect this evidence heterogeneity, with most recommending against routine LMWH use while acknowledging potential benefit in selected high-risk populations, particularly those with antiphospholipid syndrome or previous severe early-onset disease. Future research should focus on biomarker-guided patient selection, optimal dosing regimens, and integration with multimodal preventive strategies to maximize therapeutic benefit while minimizing unnecessary interventions.

## 1. Introduction

Preeclampsia represents a multisystem pregnancy disorder affecting 2–8% of gestations worldwide, manifesting as new-onset hypertension combined with proteinuria or maternal organ dysfunction after the 20th gestational week [1,2]. This condition continues to pose substantial challenges in maternal-fetal medicine, contributing significantly to global maternal and perinatal mortality rates, with disproportionate burden in resource-limited settings [3]. Maternal complications encompass eclamptic seizures, cerebrovascular accidents, multiorgan failure, and death, while fetal adverse outcomes include intrauterine growth restriction, iatrogenic prematurity, and increased perinatal mortality [4].

The underlying pathophysiology involves a biphasic process characterized by initial defective placentation followed by maternal systemic responses [5]. During early pregnancy, inadequate trophoblastic invasion results in incomplete spiral artery remodeling, creating placental hypoperfusion and oxidative stress [6]. Subsequently, placental-derived factors released into maternal circulation trigger widespread endothelial dysfunction, systemic inflammation, and clinical manifestations [7].

Evidence suggests the presence of enhanced coagulation activation and microvascular thrombosis in preeclamptic pregnancies [8]. These observations have generated interest in anticoagulant therapies as potential preventive interventions, particularly low-molecular-weight heparin (LMWH) [9]. LMWH comprises depolymerized heparin fragments ranging from 4000 to 6500 daltons molecular weight [10]. Beyond its established anticoagulant properties mediated through antithrombin-dependent factor Xa inhibition, LMWH exhibits anti-inflammatory and angiogenesis-modulating activities potentially beneficial in preeclampsia pathophysiology [11,12].

The therapeutic potential of LMWH in preeclampsia prevention encompasses mechanisms beyond coagulation modification, including enhancement of trophoblastic function, placental vascular development, and inflammatory pathway modulation [13]. Furthermore, LMWH treatment influences multiple preeclampsia-associated biomarkers, including angiogenic mediators, inflammatory cytokines, and endothelial activation markers [14].

### 1.1. Clinical Classification and Disease Manifestations

Temporal classification of preeclampsia provides crucial prognostic information and pathophysiological insights [15,16,17]. Early-onset disease (before 34 weeks) typically associates with placental pathology, severe clinical complications, and hemodynamic patterns featuring reduced cardiac output with elevated vascular resistance [15]. This phenotype frequently coincides with fetal growth restriction, reflecting profound placental dysfunction. Late-onset preeclampsia (≥34 weeks), comprising approximately 70% of cases, generally presents with preserved or increased fetal growth, variable cardiac output patterns, and heterogeneous vascular resistance profiles [16,17]. Although research emphasis often focuses on preterm preeclampsia due to its severity, term disease accounts for two-thirds of cases and substantially impacts overall disease burden [18,19].

Current classification frameworks categorize preeclampsia by severity features, including blood pressure ≥ 160/110 mmHg, significant proteinuria, thrombocytopenia, hepatic dysfunction, renal impairment, pulmonary edema, neurological symptoms, or placental insufficiency signs [3,4]. This stratification guides clinical management and prognostic counseling.

Preeclampsia’s clinical spectrum reflects multiorgan endothelial dysfunction [20,21]. Cardiovascular effects manifest as hypertension resulting from increased systemic vascular resistance despite contracted intravascular volume [15]. Pulmonary involvement ranges from subclinical edema to life-threatening acute respiratory distress syndrome, driven by capillary leak and reduced oncotic pressure [16]. Cerebrovascular manifestations range from headaches to hemorrhagic stroke and posterior reversible encephalopathy syndrome, particularly with severe hypertension [15,16].

Renal effects typically present as proteinuria from glomerular endotheliosis and podocyte damage [18]. Hepatic involvement includes transaminase elevation, subcapsular hematomas, and rarely, hepatic rupture [19]. Hematological manifestations encompass consumptive thrombocytopenia, microangiopathic hemolysis, and coagulopathy [15]. Placental insufficiency causes fetal growth restriction, while metabolic derangements may produce macrosomia [16]. Iatrogenic prematurity from indicated delivery significantly contributes to neonatal morbidity [18,19]. Long-term neurodevelopmental sequelae in offspring, including cerebral palsy risk, reflect complex interactions between placental insufficiency, maternal disease severity, and delivery timing [1,2].

### 1.2. Risk Stratification and Predictive Modeling

Preeclampsia risk assessment incorporates maternal, paternal, and pregnancy-specific factors. Maternal characteristics include demographic variables (ethnicity, maternal age extremes), medical history (chronic hypertension, diabetes, past history of preeclampsia, nulliparity), current pregnancy factors (multiple gestation, assisted reproduction), clinical findings (blood pressure, body mass index), laboratory abnormalities (anemia, thrombophilia), and sonographic markers (abnormal uterine artery Doppler) [3,21].

Risk stratification enables targeted prevention strategies for high-risk populations. Traditional screening methods assess individual risk factors independently, achieving modest detection rates of approximately 40% for preterm and 35% for term preeclampsia, with 10% false-positive rates [2,3].

Contemporary multiparametric prediction models demonstrate superior performance through integrated risk assessment. First-trimester screening at 11–13 weeks optimally predicts preterm disease, while third-trimester assessment at 35–36 weeks targets term preeclampsia [3,22]. The Fetal Medicine Foundation algorithm integrates maternal factors, mean arterial pressure, uterine artery pulsatility index, and biochemical markers (PlGF, PAPP-A) [22]. This approach achieves 90% detection for early-onset preeclampsia and 75% for preterm disease, maintaining 10% screen-positive rates [3].

### 1.3. Current Prevention Approaches

Given that definitive preeclampsia treatment requires delivery, prevention strategies targeting key pathophysiological mechanisms remain paramount. Current interventions address angiogenic imbalance, endothelial dysfunction, oxidative stress, inflammation, and vasoconstriction [21].

#### 1.3.1. Pharmacological Strategies

Low-dose aspirin represents the most established preventive intervention for high-risk women [23,24]. Meta-analyses encompassing over 36,000 participants demonstrate dose-dependent risk reduction (RR 0.82, 95% CI 0.77–0.88) with concurrent decreases in maternal complications, preterm birth, growth restriction, and perinatal mortality [20].

The ASPRE trial demonstrated remarkable efficacy of aspirin 150 mg daily initiated at 11–13 weeks, achieving 62% reduction in preterm preeclampsia (OR 0.38, 95% CI 0.20–0.74) [23]. Subsequent analyses confirm effectiveness for preterm disease prevention (RR 0.62, 95% CI 0.45–0.87) when commenced before 16 weeks at doses ≥ 100 mg daily, though term preeclampsia remains unaffected [24].

Calcium supplementation effectively prevents preeclampsia in populations with inadequate dietary intake. Meta-analysis of 30 trials (*n* = 20,445) shows substantial risk reduction (RR 0.49, 95% CI 0.39–0.61), particularly among women consuming <900 mg calcium daily [25].

Additional interventions under investigation include pravastatin (pleiotropic vascular effects), folic acid supplementation (homocysteine reduction), LMWH (anticoagulant and anti-inflammatory properties), and metformin (metabolic optimization), though evidence remains preliminary [23,24,25].

#### 1.3.2. Lifestyle Modifications

Regular physical activity demonstrates protective effects against preeclampsia (OR 0.59, 95% CI 0.37–0.90) without adverse fetal outcomes [20]. Benefits require minimum 140 min weekly of moderate-intensity exercise, potentially mediated through improved placental perfusion, enhanced antioxidant capacity, reduced inflammation, and vascular function optimization [20].

Weight management strategies show promise, particularly preconceptional optimization given strong obesity-preeclampsia associations [21,26]. However, gestational weight loss interventions lack efficacy evidence and may compromise fetal growth [27].

Nutritional interventions increasingly attract research attention as modifiable preeclampsia risk factors [27]. Dietary patterns influence inflammation, oxidative stress, endothelial function, and angiogenic balance—all relevant to disease pathogenesis. Evidence supports protective effects of plant-based, whole-food dietary patterns rich in fruits, vegetables, whole grains, and lean proteins [27]. Specific nutrients demonstrating potential benefits include antioxidants, omega-3 fatty acids, vitamin D, and dietary fiber [28,29,30,31].

Dietary pattern analysis reveals superior insights compared to single-nutrient approaches. Mediterranean dietary patterns may exert promising results for reducing preeclampsia and pregnancy complications [32,33], with recent evidence linking adherence to improved assisted reproduction outcomes [34].

### 1.4. LMWH as a Preventive Measure

Among investigational pharmacological approaches, LMWH demonstrates particular promise for preventing preeclampsia in selected high-risk populations [9]. Compared to alternative anticoagulants, LMWH offers pregnancy-specific advantages including predictable pharmacokinetics, minimal monitoring requirements, and absence of transplacental passage [35]. Research interest stems from evidence of pleiotropic LMWH effects extending beyond anticoagulation to modulate multiple preeclampsia-relevant pathophysiological processes [11,12].

This comprehensive review synthesizes current evidence examining LMWH effects on preeclampsia-associated biomarkers and evaluates clinical efficacy for disease prevention in high-risk populations. Through integration of mechanistic insights with clinical trial outcomes, we aim to elucidate LMWH’s therapeutic potential and inform future research directions and clinical applications.

## 2. Pathophysiological Foundations of Preeclampsia

### 2.1. Placental Origins and Maternal Consequences

Preeclampsia pathogenesis originates from aberrant placental development during early gestation [36]. Normal placentation involves extensive trophoblast-mediated remodeling of maternal spiral arteries, transforming muscular resistance vessels into dilated capacitance channels ensuring adequate uteroplacental perfusion [37]. Preeclamptic pregnancies demonstrate deficient arterial transformation, maintaining high-resistance vascular characteristics that are inadequate for increasing gestational demands [38].

Consequent placental hypoperfusion triggers oxidative stress and release of various bioactive mediators into maternal circulation [39]. These factors initiate cascading pathophysiological events encompassing systemic inflammation, widespread endothelial activation, and angiogenic-antiangiogenic imbalance [40]. Clinical disease manifests through hypertension, proteinuria, and potential multiorgan dysfunction [41].

Placental microthrombosis represents an additional pathological feature compromising placental function in preeclampsia [42]. Enhanced coagulation activation generates increased thrombin production and fibrin deposition within placental vasculature [43,44].

### 2.2. Biomarker Profiles in Preeclampsia

Multiple biomarkers reflect underlying pathophysiological processes and provide diagnostic, predictive, and prognostic information (Table 1).

#### 2.2.1. Angiogenic Balance Markers

Disrupted angiogenic homeostasis characterizes preeclampsia pathophysiology [45]. Placental hypoxia stimulates excessive sFlt-1 production, sequestering circulating VEGF and PlGF through high-affinity binding [46]. The sFlt-1/PlGF ratio provides valuable predictive information, with elevations preceding clinical disease by weeks [47,48].

Soluble endoglin represents another antiangiogenic mediator elevated in preeclampsia, synergizing with sFlt-1 to impair endothelial function [49]. Combined sFlt-1 and sEng administration reproduces severe preeclampsia phenotypes in experimental models [50].

#### 2.2.2. Inflammatory Mediators

Exaggerated inflammatory responses characterize preeclampsia, evidenced by elevated pro-inflammatory cytokines including TNF-α, IL-6, and IL-1β [51,52]. These mediators perpetuate endothelial dysfunction and contribute to clinical manifestations [53]. CRP elevation reflects systemic inflammation and provides predictive value [54]. Simple inflammatory indices derived from complete blood count parameters offer cost-effective screening markers, including the neutrophil-to-lymphocyte ratio (NLR) and platelet-to-lymphocyte ratio (PLR) [55]. The systemic immune inflammation index (SII), calculated as (neutrophil count × platelet count)/lymphocyte count, represents a novel composite inflammatory biomarker that integrates information from three key immune cell populations and demonstrates enhanced predictive capability for preeclampsia development compared to individual inflammatory indices [55]. Elevated SII values reflect the simultaneous activation of neutrophils and platelets alongside relative lymphopenia, capturing the complex inflammatory milieu characteristic of preeclampsia pathophysiology and providing superior discriminatory performance in risk stratification models [55].

#### 2.2.3. Endothelial Activation Markers

Endothelial dysfunction represents a central pathophysiological feature reflected by multiple biomarkers. Endothelin-1, a powerful vasoconstrictor, shows significant elevation [56,57]. Adhesion molecules including sICAM-1 and VCAM-1 increase with endothelial activation [58]. ADMA accumulation inhibits nitric oxide synthesis, promoting vasoconstriction [59]. Circulating endothelial cells and microparticles directly indicate vascular injury [60].

#### 2.2.4. Hemostatic System Markers

Preeclampsia’s prothrombotic state manifests through multiple coagulation alterations [61]. Elevated thrombin-antithrombin complexes, D-dimer, and fibrin degradation products indicate accelerated thrombin generation and fibrinolysis [62,63]. Tissue factor pathway dysregulation appears through altered TF and TFPI levels [64].

PAI-1 elevation impairs fibrinolysis, promoting fibrin accumulation [65]. Platelet activation markers, including P-selectin and platelet-derived microparticles, significantly increase [66].

#### 2.2.5. Placental-Specific Markers

Various placental products serve as potential biomarkers. PP13 demonstrates altered maternal serum levels preceding clinical disease [67]. First-trimester PAPP-A reduction associates with subsequent preeclampsia development [68].

Circulating cell-free fetal DNA increases with placental apoptosis/necrosis, preceding clinical manifestations [69]. Placental extracellular vesicles carry pathogenic factors and represent emerging biomarker candidates [70].

## 3. Pharmacology of Low-Molecular-Weight Heparin in Pregnancy

### 3.1. LMWH Preparations and Properties

LMWH production involves controlled depolymerization of unfractionated heparin, yielding polysaccharide fragments averaging 4000–6500 daltons [71]. This molecular modification enhances bioavailability, extends duration of action, improves dose–response predictability, and reduces adverse effects including thrombocytopenia and osteoporosis compared to unfractionated heparin [72].

Multiple LMWH formulations exist with distinct characteristics based on manufacturing methods (Table 2). Common obstetric preparations include enoxaparin, dalteparin, tinzaparin, nadroparin, and bemiparin [73]. Anti-Xa:anti-IIa activity ratios vary between preparations, potentially influencing non-anticoagulant effects [74].

### 3.2. Pharmacokinetics and Pharmacodynamics in Pregnancy

Gestational physiology significantly alters LMWH pharmacokinetics through expanded plasma volume, enhanced renal elimination, and modified protein binding [82,83,84]. These changes typically reduce peak anti-Xa concentrations and shorten elimination half-lives compared to non-pregnant states [83,84].

Progressive volume expansion and increased glomerular filtration throughout pregnancy affect LMWH distribution and clearance [85]. Some experts advocate weight-based dosing with potential gestational adjustments for therapeutic anticoagulation [86]. However, preeclampsia prevention trials typically utilize fixed prophylactic doses [87].

LMWH’s primary anticoagulant mechanism involves antithrombin-mediated factor Xa inhibition with lesser anti-IIa activity [88]. Anti-Xa levels provide pharmacodynamic monitoring when indicated, though prophylactic dosing rarely requires surveillance [89].

Important non-anticoagulant LMWH effects relevant to preeclampsia prevention include anti-inflammatory actions, growth factor modulation, complement inhibition, and trophoblast function enhancement [90,91,92].

### 3.3. Safety Profile in Pregnancy

LMWH’s high molecular weight and anionic charge prevent placental transfer, ensuring fetal safety during pregnancy [93]. High-quality clinical evidence confirms favorable maternal-fetal safety profiles [94].

Injection site reactions represent the most frequent adverse effect, affecting up to 40% of users but remaining generally mild [95]. Major hemorrhage risk with prophylactic dosing remains low at 0.43% [96]. Hypersensitivity reactions occur rarely but may require alternative LMWH preparations or fondaparinux substitution [97].

HIT incidence remains below 0.1% in pregnancy [98]. Osteoporosis risk is minimal compared to unfractionated heparin, with symptomatic disease affecting <1% [99].

Peripartum management requires LMWH discontinuation 24 h before planned delivery to minimize bleeding risk and permit neuraxial anesthesia [100]. Spontaneous labor necessitates delaying regional anesthesia until 24 h post-dose [101].

Breastfeeding compatibility is excellent since LMWH’s molecular size prevents milk secretion and oral absorption precludes infant exposure [102].

## 4. Effects of LMWH on Preeclampsia Biomarkers

### 4.1. Impact on Angiogenic Factors

The imbalance between pro-angiogenic and anti-angiogenic factors is a central feature of preeclampsia pathophysiology. LMWH is shown to influence this balance through various mechanisms [102].

In vitro studies have demonstrated that LMWH can bind to and modulate the activity of sFlt-1, potentially reducing its anti-angiogenic effects [103]. Heparin and its derivatives bind to the heparin-binding domain of VEGF and PlGF, potentially protecting these growth factors from sFlt-1 antagonism [104]. Additionally, LMWH induces the release of TFPI, which can displace VEGF from sFlt-1 complexes, thereby increasing free VEGF availability [104].

Several clinical studies have investigated the effects of LMWH on angiogenic markers in pregnant women at high risk for preeclampsia. Rodger et al. [105] reported that prophylactic dalteparin treatment was associated with lower sFlt-1 levels and higher PlGF levels compared to controls. Similarly, Abheiden et al. [106] found that enoxaparin treatment in women with previous preeclampsia resulted in lower sFlt-1/PlGF ratios compared to untreated controls.

However, not all studies have demonstrated significant effects of LMWH on angiogenic markers. In a randomized controlled trial by Groom et al. [107], enoxaparin treatment did not significantly alter sFlt-1 or PlGF levels compared to standard care in women at high risk for preeclampsia. These inconsistent findings may reflect differences in study populations, LMWH preparations, dosing regimens, or timing of intervention.

### 4.2. Effects on Inflammatory Markers

LMWH possesses anti-inflammatory properties that may contribute to its potential preventive effects in preeclampsia [108,109,110]. These effects include inhibition of leukocyte adhesion and transmigration, reduction in pro-inflammatory cytokine production, and modulation of complement activation [110].

In vitro studies have shown that LMWH can inhibit TNF-α-induced expression of cell adhesion molecules in endothelial cells and reduce the production of IL-6 and IL-8 in trophoblast cultures [111]. Additionally, LMWH has been shown to inhibit complement activation, particularly the alternative pathway, which is implicated in preeclampsia pathophysiology [13].

Clinical studies have provided evidence for the anti-inflammatory effects of LMWH in pregnant women. Downing et al. [111] reported that prophylactic LMWH treatment in women with previous placenta-mediated pregnancy complications was associated with reduced levels of inflammatory cytokines, including TNF-α and IL-6. Similarly, Rey et al. [14] observed lower CRP levels in LMWH-treated women compared to controls.

The effects of LMWH on neutrophil and platelet activation, which contribute to systemic inflammation in preeclampsia, have also been investigated. Studies have demonstrated that LMWH can reduce neutrophil adhesion, degranulation, and neutrophil extracellular trap formation [112]. Additionally, LMWH has been shown to inhibit platelet activation and reduce the release of platelet-derived inflammatory mediators [113].

### 4.3. Influence on Coagulation Parameters

The anticoagulant effects of LMWH are well-established and primarily mediated through its interaction with antithrombin, leading to enhanced inhibition of factor Xa and, to a lesser extent, thrombin [114]. In preeclampsia, the restoration of normal coagulation parameters may contribute to improved placental perfusion and reduced microthrombosis [115].

Clinical studies have demonstrated that prophylactic LMWH treatment in high-risk pregnancies normalizes several coagulation parameters that are altered in preeclampsia [116]. These include reductions in thrombin generation, D-dimer levels, and thrombin-antithrombin complexes [117]. Additionally, LMWH has been shown to reduce circulating tissue factor activity and increase TFPI levels, potentially modulating the extrinsic coagulation pathway that is activated in preeclampsia [118].

The effects of LMWH on fibrinolysis have also been investigated, with studies showing that LMWH can increase tissue plasminogen activator (tPA) levels and reduce PAI-1 activity [119]. These changes may enhance fibrinolytic capacity and counteract the impaired fibrinolysis observed in preeclampsia [120].

### 4.4. Effects on Placental Development and Function

LMWH may influence placental development and function through several mechanisms, including effects on trophoblast invasion, apoptosis, and placental vascularization [121].

In vitro studies have demonstrated that LMWH can enhance trophoblast invasion and migration, potentially improving spiral artery remodeling [122]. This effect appears to be mediated through increased matrix metalloproteinase (MMP) expression and activity, as well as enhanced insulin-like growth factor binding protein-1 (IGFBP-1) signaling [123].

LMWH has also been shown to reduce trophoblast apoptosis under hypoxic conditions, which may enhance placental development in the setting of placental ischemia [124]. Additionally, LMWH treatment appears to increase syncytialization and hormone production by trophoblast cells, suggesting positive effects on placental function [125].

Animal studies have provided evidence for improved placental vascularization with LMWH treatment in models of preeclampsia [126]. This effect may be mediated through enhanced expression of angiogenic factors, including VEGF and angiopoietin-1, as well as reduced expression of anti-angiogenic factors such as sFlt-1 [127].

The effects of LMWH on preeclampsia-related biomarkers are systematically presented in Table 3, which summarizes the current evidence base for LMWH’s multifaceted mechanisms of action.

**Figure 1:**
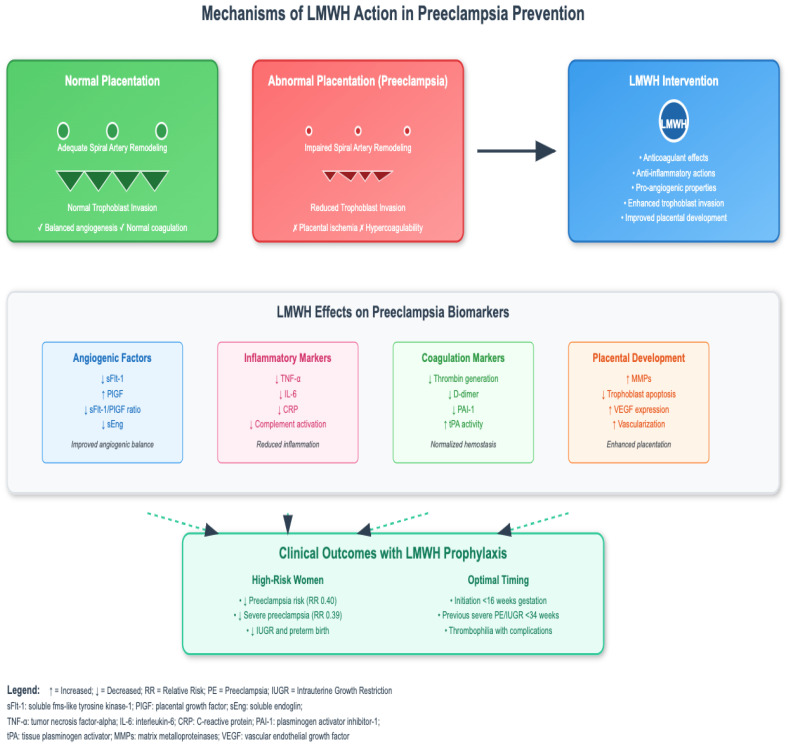
LMWH action and mechanisms in preeclampsia prevention.

The multifaceted mechanisms by which LMWH may prevent preeclampsia are summarized in Figure 1, which illustrates how LMWH’s pleiotropic actions target the key pathophysiological processes underlying preeclampsia. As depicted, LMWH intervention addresses abnormal placentation through four primary mechanisms: restoration of angiogenic balance, attenuation of inflammatory responses, normalization of coagulation parameters, and enhancement of placental development. These mechanistic effects translate into clinical benefits when LMWH is initiated early in pregnancy in carefully selected high-risk populations.

## 5. LMWH for Prevention of Preeclampsia: Clinical Evidence

### 5.1. Randomized Controlled Trials

Numerous randomized controlled trials (RCTs) have evaluated the efficacy of LMWH in preventing preeclampsia and related adverse outcomes in high-risk pregnancies, with findings systematically summarized in Table 4. These trials have varied in their inclusion criteria, LMWH preparations, dosing regimens, timing of initiation, and outcome definitions, contributing to heterogeneous results.

One of the earliest RCTs by Rey et al. [14] demonstrated a significant reduction in preeclampsia (2.8% vs. 31.3%) with dalteparin in women with previous severe placenta-mediated pregnancy complications. Similarly, Gris et al. [129] reported that enoxaparin significantly reduced preeclampsia compared to aspirin alone in women with inherited thrombophilia and previous pregnancy loss.

In contrast, several larger trials have failed to demonstrate a significant benefit of LMWH in preventing preeclampsia. The FRUIT trial by de Vries et al. [130] found no significant difference in recurrent hypertensive disorders between aspirin plus nadroparin versus aspirin alone in women with previous early-onset hypertensive disorders and thrombophilia. Similarly, the TIPPS trial by Rodger et al. [87] showed no significant reduction in recurrent placenta-mediated pregnancy complications with dalteparin in women with previous placenta-mediated complications or thrombophilia.

More recently, the HEPEPE trial by Haddad et al. [131] reported no significant effect of enoxaparin on the incidence of preeclampsia in women with previous severe preeclampsia. In contrast, the EPPI trial by Groom et al. [107] demonstrated a significant reduction in preeclampsia with enoxaparin in women at high risk based on screening and placental assessment.

These inconsistent findings may be attributed to several factors, including differences in study populations, varying definitions of high risk, differences in LMWH preparations and dosing, timing of intervention initiation, and concurrent use of aspirin [136]. Additionally, most trials have been underpowered to detect differences in relatively rare outcomes, highlighting the need for larger, well-designed studies [137].

### 5.2. Meta-Analyses and Systematic Reviews

Several meta-analyses and systematic reviews have attempted to synthesize the evidence from individual trials on LMWH for preeclampsia prevention, with key findings systematically presented in Table 5. These analyses have largely focused on specific high-risk populations and have yielded varying conclusions.

An early meta-analysis by Dodd et al. [138] concluded that LMWH reduces the risk of recurrent preeclampsia (RR 0.52, 95% CI 0.32–0.86) in women with previous severe preeclampsia or IUGR. Similarly, Rodger et al. [105] reported a significant reduction in recurrent placenta-mediated complications with LMWH in women with previous adverse pregnancy outcomes (RR 0.52, 95% CI 0.32–0.86).

A comprehensive meta-analysis by Roberge et al. [133] demonstrated that LMWH reduces the risk of preeclampsia (RR 0.40, 95% CI 0.27–0.60) and severe preeclampsia (RR 0.39, 95% CI 0.26–0.58) in high-risk women. Subgroup analyses revealed that the preventive effect was more pronounced when LMWH was initiated before 16 weeks of gestation and in women with previous placenta-mediated complications.

More recently, a Cochrane review by Skeith et al. [137] concluded that LMWH may reduce the risk of preeclampsia in women with a history of placenta-mediated complications (RR 0.46, 95% CI 0.29–0.73). However, the authors noted substantial heterogeneity among trials and highlighted the need for further high-quality studies.

### 5.3. Subgroup Analyses and Specific Populations

The inconsistent results from clinical trials and meta-analyses have prompted investigations into specific subgroups that might benefit most from LMWH prophylaxis. Several factors have been identified as potential modifiers of LMWH effectiveness in preventing preeclampsia.

#### 5.3.1. Previous Placenta-Mediated Complications

Women with a history of severe preeclampsia, particularly early-onset preeclampsia, appear to derive greater benefit from LMWH prophylaxis [144]. A meta-analysis of individual patient data by Rodger et al. [142] found that LMWH significantly reduced recurrent placenta-mediated complications in women with a history of severe or early-onset preeclampsia (RR 0.46, 95% CI 0.29–0.73).

Similarly, women with previous intrauterine growth restriction may benefit from LMWH prophylaxis, particularly when the growth restriction was severe or early-onset [145]. A subgroup analysis from the HAPPY trial demonstrated a significant reduction in recurrent IUGR with nadroparin in women with a history of severe IUGR [132].

#### 5.3.2. Thrombophilia Status

The presence of hereditary or acquired thrombophilia may influence the effectiveness of LMWH in preventing preeclampsia [146]. Some studies have suggested greater benefit in women with thrombophilia, particularly high-risk thrombophilias such as antiphospholipid syndrome, factor V Leiden homozygosity, or combined thrombophilias [147].

A meta-analysis by Skeith et al. [137] reported a more pronounced effect of LMWH in preventing recurrent placenta-mediated complications in women with thrombophilia compared to those without thrombophilia. However, other analyses have found no significant interaction between thrombophilia status and LMWH effectiveness [148].

#### 5.3.3. Timing of Intervention

The timing of LMWH initiation appears to be a critical factor influencing its effectiveness in preventing preeclampsia [149]. Subgroup analyses from meta-analyses have consistently demonstrated greater benefit when LMWH is initiated earlier in pregnancy, ideally before 16 weeks of gestation [150]. This timing corresponds to the period of placental development and spiral artery remodeling, suggesting that LMWH may exert its preventive effects by modulating early placentation processes [151]. Later initiation may be less effective, as the pathophysiological processes leading to preeclampsia may already be established [152].

## 6. Integration with Other Preventive Strategies

The optimal approach to preeclampsia prevention likely involves integration of LMWH with other evidence-based interventions rather than reliance on any single therapeutic modality. Current evidence supports a multi-modal strategy that combines pharmacological, nutritional, and lifestyle interventions tailored to individual risk profiles and clinical circumstances [153]. This section examines the potential synergies, safety considerations, and implementation strategies for combining LMWH with established preventive measures.

### 6.1. LMWH and Low-Dose Aspirin

The combination of LMWH and low-dose aspirin represents the most extensively studied dual pharmacological approach for preeclampsia prevention, with compelling theoretical rationale based on complementary mechanisms of action. Low-dose aspirin (75–150 mg daily) prevents preeclampsia primarily through irreversible inhibition of cyclooxygenase-1 in platelets, reducing thromboxane A2 production while preserving prostacyclin synthesis by endothelial cells [23,24]. This mechanism complements LMWH’s anticoagulant and pleiotropic effects, creating a comprehensive approach targeting both thrombotic and inflammatory pathways implicated in preeclampsia pathogenesis [11,12].

Meta-analytic evidence from the ASPRE trial demonstrated that low-dose aspirin (150 mg daily) initiated at 11–13 weeks’ gestation reduced preterm preeclampsia by 62% (OR 0.38, 95% CI 0.20–0.74) [23,24]. When combined with LMWH in high-risk populations, several studies have reported enhanced protective effects [144]. Saccone et al. conducted a meta-analysis of 10 randomized controlled trials (*n* = 1089) comparing LMWH plus aspirin versus aspirin alone in women with previous preeclampsia, demonstrating superior efficacy of combination therapy (OR 0.53, 95% CI 0.28–0.99) [139].

The pathophysiological rationale for combination therapy extends beyond simple additive effects. Aspirin’s antiplatelet action may enhance LMWH’s effects on placental microcirculation by reducing platelet aggregation and microthrombus formation [23]. Conversely, LMWH’s anti-inflammatory properties may augment aspirin’s cardiovascular protective effects through complementary modulation of inflammatory cascades [11,90]. Recent evidence suggests that the combination may be particularly beneficial for women with inherited thrombophilias, where both anticoagulant and antiplatelet effects address distinct pathophysiological mechanisms [132,146].

Clinical implementation of combination therapy requires careful consideration of bleeding risks and optimal dosing strategies. The safety profile of combined LMWH and aspirin appears acceptable in pregnancy, with major bleeding rates remaining below 1% in most studies [96,144]. However, peripartum management becomes more complex, requiring coordinated discontinuation strategies to minimize hemorrhagic complications while maintaining therapeutic efficacy [100,101].

### 6.2. LMWH and Calcium Supplementation

Calcium supplementation represents another evidence-based preventive intervention that may synergistically enhance LMWH efficacy through distinct but complementary mechanisms. Meta-analyses demonstrate that calcium supplementation (1000–2000 mg daily) reduces preeclampsia risk by approximately 50% (RR 0.49, 95% CI 0.39–0.61), particularly among women with low dietary calcium intake [25]. The protective mechanism involves maintenance of normal vascular smooth muscle function, reduced parathyroid hormone release, and enhanced nitric-oxide-mediated vasodilation [154,155].

The theoretical basis for combining LMWH with calcium supplementation rests on their complementary effects on vascular function and placental development. While LMWH primarily addresses coagulation activation and inflammatory processes, calcium supplementation directly influences vascular reactivity and blood pressure regulation [25,154]. This combination may be particularly beneficial in populations with habitually low calcium intake, where dietary deficiency compounds the vascular dysfunction associated with preeclampsia pathogenesis [155].

Emerging evidence from nutritional intervention studies suggests that the combination approach may offer superior protection compared to either intervention alone. The DASH (Dietary Approaches to Stop Hypertension) diet, which naturally provides increased calcium through low-fat dairy products while emphasizing other nutrients complementary to LMWH action, presents significant reductions in preeclampsia risk when implemented during pregnancy [156,157]. Women with the highest adherence to DASH dietary patterns showed 35–45% lower preeclampsia rates compared to those with minimal adherence [156].

The integration of LMWH with calcium supplementation requires attention to timing and dosing considerations. Calcium supplementation should ideally begin during preconception care or early pregnancy to optimize vascular adaptations, while LMWH initiation before 16 weeks’ gestation appears most effective for preventing early-onset disease [149,150]. Combined therapy appears safe, with no significant drug interactions or enhanced adverse effects reported in available studies [25,154].

### 6.3. LMWH and Lifestyle Interventions

Lifestyle modifications, including regular physical activity, weight management, and dietary optimization, represent fundamental components of preeclampsia prevention that may significantly enhance LMWH efficacy [20,158]. The physiological benefits of exercise during pregnancy—improved placental perfusion, enhanced antioxidant defenses, reduced inflammation, and optimized endothelial function—complement LMWH’s anti-inflammatory and vascular protective effects [20,158].

Meta-analyses demonstrate that regular moderate-intensity exercise (≥140 min weekly) reduces preeclampsia risk by approximately 40% (OR 0.59, 95% CI 0.37–0.90) without adverse fetal effects [20]. When combined with LMWH in high-risk populations, preliminary evidence suggests enhanced protective benefits. The study by Vesco et al. examined a combined intervention of DASH dietary patterns, physical activity, and weight management in obese pregnant women, though LMWH was not included in their protocol [159]. However, the pathophysiological rationale supports potential synergy between LMWH and structured lifestyle interventions.

Weight management represents a particularly important consideration, given the strong association between maternal obesity and preeclampsia risk [158,160]. Obese women demonstrate a two- to three-fold increased preeclampsia risk compared to normal-weight women, mediated through chronic inflammation, insulin resistance, and oxidative stress—pathways that LMWH may help attenuate [160]. Pre-pregnancy weight optimization combined with LMWH prophylaxis during pregnancy may offer superior protection compared to either intervention alone [158].

Dietary interventions beyond calcium supplementation may enhance LMWH effectiveness through multiple mechanisms. The Mediterranean dietary pattern, emphasizing fruits, vegetables, whole grains, and omega-3 fatty acids, has demonstrated protective effects against preeclampsia through anti-inflammatory and antioxidant pathways [33,161]. Recent evidence suggests that higher adherence to Mediterranean dietary patterns during pregnancy is associated with reduced preeclampsia risk and improved cardiovascular outcomes [161]. The combination of Mediterranean or DASH dietary principles with LMWH prophylaxis represents a promising approach that warrants formal investigation [156,161].

Stress reduction techniques, while less extensively studied, may complement LMWH therapy by addressing psychological factors contributing to hypertensive disorders. Chronic stress activates inflammatory pathways and sympathetic nervous system responses that LMWH’s anti-inflammatory properties may help counteract [162]. Mindfulness-based interventions and stress management techniques during pregnancy have shown preliminary benefits for blood pressure control and may enhance the effectiveness of pharmacological interventions [162].

### 6.4. Risk-Stratified Combination Approaches

The heterogeneity of preeclampsia pathogenesis and varying patient risk profiles necessitate individualized combination strategies rather than universal approaches [3,22]. Risk stratification models, such as the Fetal Medicine Foundation algorithm incorporating maternal characteristics, mean arterial pressure, uterine artery pulsatility index, and biochemical markers (PlGF, PAPP-A), achieve 90% detection rates for early-onset preeclampsia while maintaining 10% screen-positive rates [22]. These prediction models enable targeted deployment of combination interventions to women most likely to benefit.

For women at highest risk—those with previous severe early-onset preeclampsia, significant thrombophilia, or multiple risk factors—comprehensive combination therapy including LMWH, low-dose aspirin, calcium supplementation, and lifestyle interventions may be warranted [3,22]. This intensive approach addresses the multiple pathophysiological pathways contributing to disease development while maximizing protective effects. Meta-analytic evidence supports enhanced efficacy of multi-modal approaches in high-risk populations, with relative risk reductions of 50–70% achievable through comprehensive interventions [141,142].

Intermediate-risk women, including those with moderate thrombophilia, chronic hypertension, or obesity, may benefit from selective combination approaches tailored to their specific risk profile [3]. For example, women with chronic hypertension might receive LMWH plus low-dose aspirin and lifestyle interventions, while those with thrombophilia might receive LMWH plus calcium supplementation [146,155]. This targeted approach optimizes the risk–benefit ratio while minimizing unnecessary interventions and associated costs.

Low-risk women with single risk factors or mild elevations in biomarkers may benefit from single-agent prophylaxis with careful monitoring for disease progression [3]. However, the threshold for initiating combination therapy should remain low given the potential for rapid disease progression and the acceptable safety profile of most preventive interventions [22].

Biomarker-guided approaches represent an emerging strategy for optimizing combination therapy selection and timing. Elevated sFlt-1/PlGF ratios, abnormal uterine artery Doppler findings, or elevated inflammatory markers may guide intensification of preventive measures [47,163]. Women demonstrating early biomarker abnormalities might benefit from immediate initiation of combination therapy, while those with normal biomarkers could receive standard preventive measures with enhanced monitoring [163,164].

**Figure 2:**
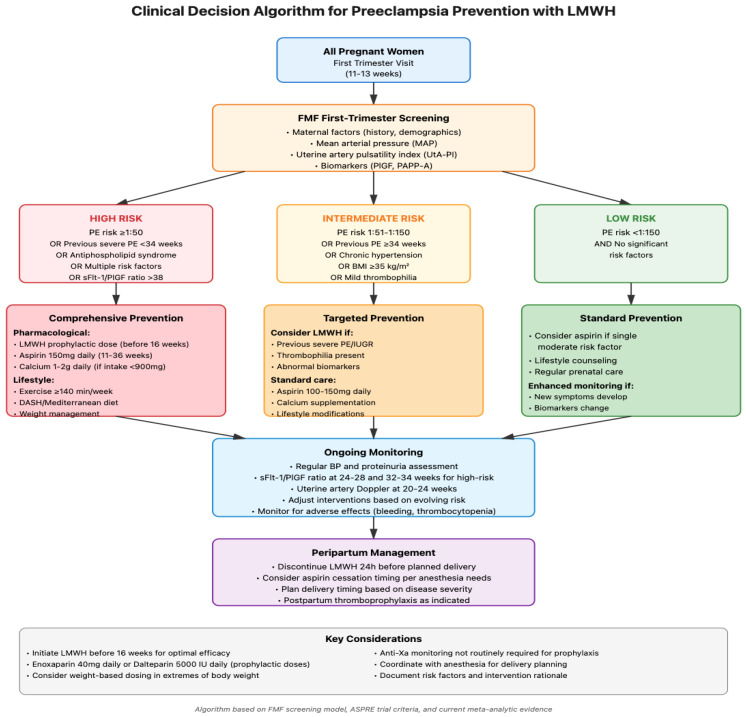
Clinical decision-making algorithm for risk-stratified preeclampsia prevention incorporating LMWH.

The implementation of risk-stratified preventive strategies requires a systematic approach that integrates validated screening algorithms with evidence-based interventions. Figure 2 presents a clinical decision-making flowchart that operationalizes the risk-stratified approach to preeclampsia prevention, incorporating the Fetal Medicine Foundation (FMF) first-trimester screening algorithm with targeted pharmacological and lifestyle interventions. This algorithm emphasizes early risk assessment at 11–13 weeks’ gestation, when interventions demonstrate maximal efficacy, and provides clear guidance for clinicians regarding the selection and timing of preventive measures based on individual risk profiles.

### 6.5. Timing and Sequencing Considerations

The optimal timing and sequencing of combination interventions represents a critical factor influencing preventive efficacy, with evidence suggesting that earlier initiation generally provides superior outcomes [149,150]. Preconceptional intervention may offer maximal benefits by optimizing maternal health status before pregnancy and ensuring optimal early placentation [165]. Women with previous severe preeclampsia or significant thrombophilia should ideally receive comprehensive preconceptional counseling addressing weight optimization, dietary modifications, and consideration of early LMWH initiation [165].

First-trimester intervention (6–16 weeks) represents the optimal window for most preventive strategies, coinciding with critical periods of placental development and spiral artery remodeling [149,150]. LMWH initiation before 16 weeks appears most effective for preventing early-onset disease, while aspirin demonstrates maximal efficacy when started before 16 weeks at doses ≥ 100 mg daily [24,149]. Calcium supplementation and lifestyle interventions should ideally commence during this period to influence fundamental placental development processes [25,166].

The sequencing of interventions may influence overall effectiveness and patient adherence. Initiating lifestyle modifications and nutritional interventions during preconception or early pregnancy establishes healthy behavioral patterns that support subsequent pharmacological interventions [166,167]. LMWH and aspirin can then be introduced as indicated based on risk assessment and biomarker findings, with timing optimized according to individual risk profiles [149,150].

Monitoring strategies must be adapted to accommodate combination therapy, with particular attention to biomarker trends and clinical responses that might guide therapy intensification or modification [158,168]. Women receiving combination therapy require enhanced surveillance for both efficacy and safety endpoints, including regular assessment of blood pressure, proteinuria, biomarker levels, and potential adverse effects [3,22].

### 6.6. Safety Considerations in Combination Therapy

The safety profile of combination preventive strategies requires careful evaluation, particularly regarding bleeding risks, drug interactions, and cumulative adverse effects [169]. While individual interventions (LMWH, aspirin, calcium supplementation) demonstrate acceptable safety profiles in pregnancy, their combination may theoretically increase certain risks, particularly hemorrhagic complications [96,169].

Bleeding risk assessment must consider both maternal and fetal factors, including gestational age, concomitant medications, underlying medical conditions, and planned delivery approach [100,101]. The combination of LMWH and aspirin increases theoretical bleeding risk through complementary anticoagulant and antiplatelet effects, though clinical studies suggest acceptable safety profiles with appropriate monitoring and peripartum management [144,169]. Major bleeding rates remain below 1% in most studies of combination therapy, comparable to rates observed with single-agent prophylaxis [96,144].

Peripartum management becomes increasingly complex with combination therapy, requiring coordinated discontinuation strategies and contingency planning for emergency delivery [100,101]. LMWH should be discontinued 24 h before planned delivery or upon onset of active labor, while aspirin cessation timing depends on individual risk assessment and anesthetic requirements [100,101]. Clear protocols must be established for managing anticoagulation reversal if emergency surgery becomes necessary [101].

Drug interactions require consideration, particularly in women receiving multiple medications for comorbid conditions [169]. Calcium supplementation may affect absorption of other medications, necessitating temporal separation of administration. Iron supplementation, commonly prescribed during pregnancy, may require dose adjustment when combined with calcium due to competitive absorption [170]. Healthcare providers must maintain comprehensive medication reconciliation and monitor for potential interactions throughout pregnancy.

Fetal safety considerations include potential effects on growth, development, and perinatal outcomes [102,171]. Available evidence suggests that LMWH, aspirin, and calcium supplementation do not adversely affect fetal development when used appropriately [93,102]. However, long-term follow-up studies of offspring exposed to combination therapy during pregnancy remain limited, representing an important area for future investigation [171].

Cost-effectiveness analyses become increasingly important with combination approaches, as the aggregate expense of multiple interventions must be justified by demonstrable improvements in outcomes [172,173]. While individual cost-effectiveness analyses support the use of aspirin and calcium supplementation in high-risk populations, comprehensive economic evaluations of combination strategies are needed to guide healthcare policy and resource allocation [173,174].

## 7. Guidelines and Current Recommendations

### International Guidelines

Several international organizations have issued guidelines regarding the use of LMWH for preeclampsia prevention in high-risk pregnancies, with recommendations comprehensively outlined in Table 6. These recommendations vary in their specificity and strength, reflecting the uncertain evidence base and the need for individualized risk assessment approaches.

The American College of Obstetricians and Gynecologists (ACOG) does not specifically recommend LMWH for preeclampsia prevention in its guidelines on hypertensive disorders of pregnancy [175]. However, their thrombophilia guidelines acknowledge that LMWH may be considered in women with antiphospholipid syndrome and previous adverse pregnancy outcomes, including preeclampsia [176].

The Society of Obstetricians and Gynaecologists of Canada (SOGC) suggests that LMWH may be considered for women with previous placenta-mediated complications, particularly those with previous severe preeclampsia and/or fetal growth restriction before 34 weeks of gestation [177]. Similarly, they recognize the potential benefit in women with thrombophilia and previous placenta-mediated complications [178].

The Royal College of Obstetricians and Gynaecologists (RCOG) recommends considering LMWH prophylaxis in women with antiphospholipid syndrome and previous adverse pregnancy outcomes, including preeclampsia [179]. However, they do not routinely recommend LMWH for preeclampsia prevention in the absence of thrombophilia [180].

The International Society for the Study of Hypertension in Pregnancy (ISSHP) explicitly recommends against the use of LMWH for preeclampsia prevention in their 2021 guidelines, providing strong evidence-based recommendations (⊕⊕⊕O/Strong) that women should not receive low-molecular-weight heparin for preeclampsia prevention [22]. However, they acknowledge that this recommendation specifically relates to preeclampsia prevention and does not preclude LMWH use for other indications, such as thromboprophylaxis in antiphospholipid antibody syndrome [22].

## 8. Limitations and Areas for Future Directions

### 8.1. Current Evidence Limitations and Research Gaps

Despite promising mechanistic rationale, several critical limitations constrain current evidence for LMWH in preeclampsia prevention. The heterogeneity in trial results partly reflects inconsistent definitions of “high-risk” populations and variable LMWH dosing regimens across studies [136]. A primary limitation is the lack of large, adequately powered randomized trials specifically designed with biomarker stratification. Current studies have been largely underpowered for subgroup analyses based on validated biomarker profiles such as sFlt-1/PlGF ratios, inflammatory markers, or coagulation parameters [141,142]. Most trials enrolled fewer than 300 participants, limiting detection of clinically meaningful differences within biomarker-defined subgroups [143,148].

Current research lacks harmonized definitions of risk categories, standardized inclusion criteria, and consensus on optimal LMWH preparations and dosing strategies. This methodological inconsistency prevents meaningful comparison of results and complicates meta-analyses, contributing to substantial heterogeneity observed in systematic reviews [143,148]. International collaboration is essential to develop unified protocols that enable robust evidence synthesis.

### 8.2. Ethical and Cost-Effectiveness Considerations

Broader implementation of LMWH prophylaxis raises important ethical and economic considerations. Ethical considerations include the balance between potential benefits and risks of anticoagulation during pregnancy, informed consent processes for investigational use, and equitable access to interventions [185]. The cost-effectiveness of extended LMWH therapy must be rigorously evaluated against alternative preventive strategies, particularly in resource-limited settings [186]. Cost-effectiveness analyses suggest that targeted approaches, focusing on women at highest risk, may provide better value than universal application [187]. Healthcare systems must develop frameworks for appropriate patient selection to optimize resource allocation while ensuring evidence-based clinical decisions.

### 8.3. Priority Areas for Future Research

#### 8.3.1. Biomarker-Guided Trial Design

Future trials should incorporate prospective biomarker stratification with adequate sample sizes to detect clinically meaningful differences within biomarker-defined subgroups [164]. Angiogenic markers, particularly the sFlt-1/PlGF ratio, may identify women with early angiogenic imbalance who might benefit from LMWH’s pro-angiogenic effects [188]. Studies showed that women with elevated sFlt-1/PlGF ratios in early pregnancy have higher preeclampsia risk and may show greater biomarker improvement with LMWH treatment [189].

Markers of hypercoagulability, including D-dimer, thrombin generation parameters, and factor VIII levels, may identify women with activated coagulation who may benefit from LMWH’s anticoagulant effects [190]. Preliminary studies suggest women with elevated D-dimer levels in early pregnancy may derive greater benefit from LMWH prophylaxis [191].

#### 8.3.2. Dose-Optimization and Timing Studies

Priority investigations include dose-optimization studies examining weight-adjusted versus fixed-dose regimens, given physiological changes in LMWH pharmacokinetics during pregnancy [84]. Optimal timing requires investigation, with studies comparing preconception, first trimester, and later pregnancy initiation strategies [149,150]. Evidence consistently demonstrates greater benefit when LMWH is initiated before 16 weeks’ gestation.

#### 8.3.3. Novel Combination Therapies

Future research should explore synergies between LMWH and emerging agents such as complement inhibitors, statins, or metformin [23,24,25]. Meta-analytic evidence demonstrates relative risk reductions of 50–70% achievable through comprehensive interventions in high-risk populations [141,142]. Combination therapy trials examining LMWH plus aspirin, calcium supplementation, or lifestyle interventions could identify synergistic approaches maximizing preventive benefits [139,192].

#### 8.3.4. Long-Term Outcome Assessment

Long-term follow-up studies are essential to evaluate effects of prenatal LMWH exposure on maternal cardiovascular health and offspring neurodevelopment [79,193]. Whether LMWH prophylaxis influences long-term maternal cardiovascular outcomes or offspring development remains unknown and represents important research priorities.

#### 8.3.5. Standardization Initiatives

Standardization of outcome measures and definitions is essential to improve comparability and enable robust meta-analyses [148]. International consensus regarding standardized definitions, inclusion criteria, and outcome measures would substantially improve research quality. Population-specific trials are needed to address varying effectiveness across different ethnic groups, BMI categories, and comorbidity profiles [164].

### 8.4. Implementation Challenges and Solutions

Implementation faces several challenges, including patient acceptance, resource constraints, and healthcare system barriers [185]. Patient acceptance and adherence to daily LMWH injections can be challenging, with discontinuation rates of 10–20% reported [191]. Educational interventions, patient support programs, and partner involvement in injection administration improve adherence [192].

LMWH is relatively expensive, and prolonged prophylaxis throughout pregnancy incurs substantial costs [193]. Clinical decision support tools incorporating risk stratification algorithms and treatment protocols could improve implementation consistency. Healthcare provider education programs addressing evidence base, patient selection criteria, and monitoring protocols will be essential for successful implementation.

## 9. Conclusions

Low-molecular-weight heparin represents a promising intervention for preeclampsia prevention in high-risk pregnancies, with plausible mechanisms of action beyond anticoagulation that address key pathophysiological processes. The effects of LMWH on preeclampsia-related biomarkers, including angiogenic factors, inflammatory mediators, and markers of coagulation, provide a strong biological rationale for its use. However, clinical evidence for LMWH in preeclampsia prevention remains inconsistent, with some trials demonstrating significant benefit while others show no effect. This heterogeneity likely reflects differences in study populations, intervention protocols, and outcome definitions. Meta-analyses suggest that LMWH may be effective in specific high-risk subgroups, particularly women with previous severe placenta-mediated complications or thrombophilia.

Current guidelines generally do not recommend routine use of LMWH for preeclampsia prevention but suggest that it may be considered in selected high-risk women, particularly those with previous severe early-onset preeclampsia, fetal growth restriction, or antiphospholipid syndrome. A risk-stratified approach, incorporating clinical history, biomarker profiles, and placental assessment, may help identify women most likely to benefit from LMWH prophylaxis.

The integration of LMWH with other evidence-based preventive strategies, including low-dose aspirin, calcium supplementation, and lifestyle interventions, offers potential for enhanced protective effects through complementary mechanisms. Risk-stratified combination approaches may optimize the risk–benefit ratio while minimizing unnecessary interventions and associated costs. However, implementation requires careful attention to timing, sequencing, and safety considerations, particularly regarding bleeding risks and peripartum management.

Future research should focus on refining patient selection through biomarker-guided approaches, optimizing dosing regimens, and investigating novel combinations. Ongoing trials addressing these questions may provide more definitive evidence to guide clinical practice. Implementation challenges, including patient acceptance, cost considerations, and healthcare system barriers, must be addressed to translate research findings into improved outcomes for high-risk pregnancies.

In conclusion, while LMWH is not a universal solution for preeclampsia prevention, it represents a valuable option for selected high-risk women. A personalized approach, based on comprehensive risk assessment and biomarker profiling, holds promise for optimizing the risk–benefit balance of LMWH prophylaxis in preeclampsia prevention.

## Figures and Tables

**Table 1:** Key biomarkers associated with preeclampsia.

Category	Biomarkers	Alteration in Preeclampsia	Potential Clinical Utility	References
Angiogenic Factors	sFlt-1	Increased	Prediction, diagnosis, prognosis	[45,46,47]
	PlGF	Decreased	Prediction, diagnosis, prognosis	[45,46,47]
	sFlt-1/PlGF ratio	Increased	Prediction, diagnosis, rule-out	[46,47]
	sEng	Increased	Prediction, severity assessment	[48,49]
Inflammatory Markers	TNF-α	Increased	Pathophysiological understanding	[50,51]
	IL-6	Increased	Prediction, severity assessment	[50,51]
	IL-1β	Increased	Pathophysiological understanding	[22,51]
	CRP	Increased	Risk assessment	[52,53,54]
	NLR, PLR	Increased	Simple screening tools	[54]
Endothelial Dysfunction	Endothelin-1	Increased	Severity assessment	[55,56]
	sICAM-1, VCAM-1	Increased	Pathophysiological understanding	[57]
	ADMA	Increased	Risk stratification	[58]
	Endothelial microparticles	Increased	Severity assessment	[59]
Coagulation and Fibrinolysis	Thrombin–antithrombin complexes	Increased	Hypercoagulability assessment	[60,61]
	D-dimer	Increased	Hypercoagulability assessment	[60,61]
	TF, TFPI	Altered levels	Pathophysiological understanding	[62]
	PAI-1	Increased	Fibrinolytic capacity assessment	[63]
	Platelet activation markers	Increased	Platelet function assessment	[64]
Placental-Derived Factors	PP13	Altered levels	Early prediction	[65]
	PAPP-A	Decreased (1st trimester)	First-trimester screening	[66]
	Cell-free fetal DNA	Increased	Prediction, severity assessment	[67]
	Placental extracellular vesicles	Altered profile	Emerging biomarkers	[68]

sFlt-1: soluble fms-like tyrosine kinase-1; PlGF: placental growth factor; sEng: soluble endoglin; TNF-α: tumor necrosis factor-alpha; IL-6: interleukin-6; IL-1β: interleukin-1 beta; CRP: C-reactive protein; NLR: neutrophil-to-lymphocyte ratio; PLR: platelet-to-lymphocyte ratio; sICAM-1: soluble intercellular adhesion molecule-1; VCAM-1: vascular cell adhesion molecule-1; ADMA: asymmetric dimethylarginine; TF: tissue factor; TFPI: tissue factor pathway inhibitor; PAI-1: plasminogen activator inhibitor-1; PP13: placental protein 13; PAPP-A: pregnancy-associated plasma protein A.

**Table 2:** Characteristics of LMWH preparations used in pregnancy.

LMWH	Mean Molecular Weight (Daltons)	Anti-Xa:Anti-IIa Ratio	Half-Life (h)	Dosing in Pregnancy Prevention	Dosing in Pregnancy Treatment	References
Enoxaparin	4500	3.8:1	4.5–7	40 mg OD or 20 mg OD (weight < 50 kg)	1 mg/kg BID or 1.5 mg/kg OD	[74,75]
Dalteparin	6000	2.7:1	3–5	5000 IU OD	100 IU/kg BID or 200 IU/kg OD	[76,77]
Tinzaparin	6500	1.9:1	3–4	4500 IU OD	175 IU/kg OD	[78,79]
Nadroparin	4300	3.6:1	3.5–4	2850 IU OD	85.5 IU/kg BID or 171 IU/kg OD	[80]
Bemiparin	3600	8:1	5–6	2500–3500 IU OD	115 IU/kg OD	[81]

OD: once daily; BID: twice daily; IU: international units.

**Table 3:** Effects of LMWH on preeclampsia-related biomarkers.

Category	Biomarker	LMWH Effect	Proposed Mechanism	References
Angiogenic Factors	sFlt-1	Decrease	Binding and neutralization, reduced placental production	[103,104,106]
	PlGF	Increase	Protection from sFlt-1 antagonism, enhanced production	[104,106,107]
	sFlt-1/PlGF ratio	Decrease	Combined effect on sFlt-1 and PlGF	[107]
	sEng	Decrease/No effect	Variable effects reported	[103,108]
Inflammatory Markers	TNF-α	Decrease	Inhibition of production, enhanced clearance	[111,128]
	IL-6	Decrease	Reduced production by trophoblasts and immune cells	[111,128]
	CRP	Decrease	General anti-inflammatory effect	[13]
	Complement activation	Decrease	Inhibition of alternative pathway	[128]
	Leukocyte adhesion molecules	Decrease	Reduced expression on endothelial cells	[110,111]
Coagulation and Fibrinolysis	Thrombin generation	Decrease	Enhanced antithrombin activity	[116,117]
	D-dimer	Decrease	Reduced fibrin formation and degradation	[117]
	Tissue factor activity	Decrease	Direct inhibition, increased TFPI	[118]
	PAI-1	Decrease	Enhanced clearance, reduced production	[119,120]
	tPA activity	Increase	Reduced PAI-1 inhibition	[119]
Placental Development	MMPs	Increase	Enhanced expression and activity	[122,123]
	Trophoblast apoptosis	Decrease	Anti-apoptotic signaling	[124]
	Placental VEGF expression	Increase	Stimulation of production, protection from degradation	[126,127]
	Placental vascularization	Increase	Pro-angiogenic effects, reduced ischemia	[126,127]

sFlt-1: soluble fms-like tyrosine kinase-1; PlGF: placental growth factor; sEng: soluble endoglin; TNF-α: tumor necrosis factor-alpha; IL-6: interleukin-6; CRP: C-reactive protein; TFPI: tissue factor pathway inhibitor; PAI-1: plasminogen activator inhibitor-1; tPA: tissue plasminogen activator; MMPs: matrix metalloproteinases; VEGF: vascular endothelial growth factor.

**Table 4:** Key randomized controlled trials of LMWH for preeclampsia prevention.

Study (Year)	Population	Sample Size	Intervention	Control	Initiation	Primary Outcome	Preeclampsia Results	Other Outcomes	Confounders
Rey et al. (2009) [14]	Previous severe PE/IUGR/abruption/SB	110	Dalteparin 5000 IU/day + ASA	ASA	<16 weeks	Composite: PE/IUGR/abruption/SB	PE: 2.8% vs. 31.3% (*p* < 0.001)	Significant reduction in composite outcome	Small sample size, open-label design
Gris et al. (2004) [129]	Thrombophilia + previous loss	160	Enoxaparin 40 mg/day	ASA 100 mg/day	<8 weeks	Live birth	PE: 0% vs. 10.0% (*p* = 0.01)	Significant improvement in live birth rate	Selected population with thrombophilia
de Vries et al. (2012) [130] FRUIT	Previous early HDP + thrombophilia	139	Nadroparin 3800 IU/day + ASA	ASA	<12 weeks	Recurrent HDP < 34 weeks	No significant difference in recurrent PE	Earlier onset of recurrent HDP in control group	Heterogeneous thrombophilia types
Rodger et al. (2014) [87] TIPPS	Previous PE/IUGR/abruption/SB or thrombophilia	292	Dalteparin 5000 IU/day	No LMWH	<21 weeks	Composite: PE/IUGR/VTE/SB	No significant difference in PE	No significant difference in composite outcome	Mixed population with/without thrombophilia
Haddad et al. (2016) [131] HEPEPE	Previous severe PE	224	Enoxaparin 40 mg/day	No LMWH	12–16 weeks	Preeclampsia	PE: 10.4% vs. 18.9% (*p* = 0.09)	No significant differences in secondary outcomes	Late initiation of intervention
Groom et al. (2017) [107] EPPI	High risk by screening + abnormal uterine artery	149	Enoxaparin 40 mg/day	Standard care	12–14 weeks	PE/SGA < 5th percentile	PE: 8.0% vs. 22.2% (*p* = 0.02)	Significant reduction in preterm birth	Doppler-guided selection
Martinelli et al. (2017) [132] HAPPY	Previous early PE/IUGR/abruption	156	Nadroparin 3800 IU/day	No LMWH	<14 weeks	Composite: PE/IUGR/abruption/SB	No significant difference in PE	No significant difference in composite outcome	Underpowered for individual outcomes
Roberge et al. (2016) [133]	FGR history or high risk by screening	91	Dalteparin 5000 IU/day + ASA	ASA	11–14 weeks	Uterine artery PI at 22–24 weeks	PE: 2.1% vs. 17.0% (*p* = 0.03)	Significant improvement in uterine artery flow	Small sample size
McLaughlin et al. (2022) [134] HepASA	Previous placental syndrome	380	Dalteparin 5000 IU/day + ASA	ASA	6–16 weeks	Composite: PE/IUGR/abruption/SB	No significant difference in PE	No significant difference in composite outcome	Broad inclusion criteria
Seidler et al. (2019) [135]	Severe PE/FGR history or high risk	314	Enoxaparin 40 mg/day	Standard care	12–16 weeks	Composite adverse outcome	PE: 9.7% vs. 17.6% (*p* = 0.046)	Significant reduction in preterm birth < 37 weeks	Risk-stratified approach

PE: preeclampsia; IUGR: intrauterine growth restriction; SGA: small for gestational age; FGR: fetal growth restriction; SB: stillbirth; HDP: hypertensive disorders of pregnancy; VTE: venous thromboembolism; ASA: aspirin; PI: pulsatility index.

**Table 5:** Meta-analyses and systematic reviews of LMWH for preeclampsia prevention.

Study (Year)	Included Studies	Population	Intervention	Comparison	Preeclampsia Outcome	Other Outcomes	Conclusions	Confounders
Dodd et al. (2013) [138]	8 RCTs (963 women)	Previous PE/IUGR	LMWH +/− aspirin	Placebo/aspirin/standard care	RR 0.52 (95% CI 0.32–0.86)	Reduced IUGR (RR 0.54, 95% CI 0.32–0.91)	LMWH reduces recurrent PE and IUGR	Heterogeneity in study populations
Rodger et al. (2014) [105]	6 RCTs (848 women)	Previous placenta-mediated complications	LMWH	No LMWH	RR 0.47 (95% CI 0.22–1.03)	Reduced composite outcome (RR 0.52, 95% CI 0.32–0.86)	LMWH reduces recurrent placenta-mediated complications	Variable thrombophilia status
Roberge et al. (2016) [133]	8 RCTs (885 women)	High-risk pregnancies	LMWH +/− aspirin	Placebo/aspirin/standard care	RR 0.40 (95% CI 0.27–0.60)	Reduced severe PE (RR 0.39, 95% CI 0.26–0.58)	LMWH reduces PE and severe PE in high-risk women	Timing of initiation variable
Skeith et al. (2016) [137] Cochrane	9 RCTs (979 women)	Previous placenta-mediated complications	LMWH	No LMWH	RR 0.46 (95% CI 0.29–0.73)	No significant reduction in other individual outcomes	LMWH may reduce PE in women with prior complications	Substantial trial heterogeneity
Saccone et al. (2017) [139]	10 RCTs (1089 women)	Previous PE	LMWH +/− aspirin	Placebo/aspirin/standard care	LMWH + ASA vs. ASA: OR 0.53 (95% CI 0.28–0.99)	LMWH + ASA reduced composite adverse outcome vs. ASA	LMWH + ASA more effective than ASA alone for recurrent PE	Concurrent aspirin use variable
Maher et al. (2017) [140]	4 RCTs (522 women)	Previous PE	LMWH + aspirin	Aspirin	RR 0.70 (95% CI 0.40–1.23)	No significant difference in other outcomes	No added benefit of LMWH over aspirin alone	Limited to aspirin comparison
Zhang et al. (2015) [141]	11 RCTs (1115 women)	Previous placenta-mediated complications	LMWH +/− aspirin	No LMWH +/− aspirin	RR 0.42 (95% CI 0.28–0.62)	Reduced IUGR (RR 0.56, 95% CI 0.41–0.77)	LMWH reduces recurrent PE and IUGR	Dosing regimens varied
Rodger et al. (2016) [142]	21 RCTs (2876 women)	Multiple high-risk groups	LMWH +/− aspirin	No LMWH +/− aspirin	All women: RR 0.63 (95% CI 0.46–0.87)	Reduced IUGR and placental abruption	LMWH effective for specific high-risk subgroups	Population heterogeneity
Lin et al. (2023) [143]	14 RCTs (2451 women)	Previous PE/IUGR or high risk	LMWH +/− aspirin	Standard care/aspirin	RR 0.59 (95% CI 0.47–0.75)	Reduced IUGR and preterm birth	LMWH reduces PE and improves other outcomes	Study quality variable

RR: relative risk; OR: odds ratio; CI: confidence interval; PE: preeclampsia; IUGR: intrauterine growth restriction; ASA: aspirin; LMWH: low-molecular-weight heparin; RCT: randomized controlled trial.

**Table 6:** International guidelines and recommendations on LMWH for preeclampsia prevention.

Organization	Year	Recommendations	Population	Evidence Level
ACOG [175,176]	2020	No specific recommendation for LMWH for PE prevention	N/A	N/A
		May consider LMWH in APS with previous adverse outcomes	APS + previous PE/IUGR	Low
SOGC [177,178]	2019	May consider LMWH in previous severe early-onset PE/IUGR	Previous PE/IUGR < 34 weeks	Moderate
		May consider LMWH in thrombophilia with previous placental complications	Thrombophilia + previous PE/IUGR	Low
RCOG [179,180]	2018	Consider LMWH in APS with previous adverse outcomes	APS + previous PE/IUGR	Moderate
		Not routinely recommended for PE prevention without thrombophilia	Previous PE without thrombophilia	Low
ISSHP [22]	2021	Does NOT recommend LMWH for PE prevention	All women	Strong
		LMWH acceptable for other indications (e.g., thromboprophylaxis in APS)	Specific indications	Moderate
ISTH [181,182]	2020	May consider LMWH in previous severe placenta-mediated complications	Previous severe PE/IUGR	Low
		Higher priority if thrombophilia present	Thrombophilia + previous PE/IUGR	Moderate
ACCP [183,184]	2022	Does not recommend routine LMWH for PE prevention	All women at risk for PE	Low
		LMWH only if other indications for anticoagulation exist	Specific high-risk groups	Moderate

ACOG: American College of Obstetricians and Gynecologists; SOGC: Society of Obstetricians and Gynaecologists of Canada; RCOG: Royal College of Obstetricians and Gynaecologists; ISTH: International Society on Thrombosis and Haemostasis; ACCP: American College of Chest Physicians; PE: preeclampsia; IUGR: intrauterine growth restriction; APS: antiphospholipid syndrome.

## Data Availability

No new data were created or analyzed in this study. Data sharing is not applicable to this article.

## Abbreviations

The following abbreviations are used in this manuscript:

ACCP	American College of Chest Physicians
ACOG	American College of Obstetricians and Gynecologists
ADMA	Asymmetric dimethylarginine
APS	Antiphospholipid syndrome
ASA	Aspirin
BID	Twice daily
BMI	Body mass index
CI	Confidence interval
CRP	C-reactive protein
DASH	Dietary Approaches to Stop Hypertension
FGR	Fetal growth restriction
FMF	Fetal Medicine Foundation
HDP	Hypertensive disorders of pregnancy
HIT	Heparin-induced thrombocytopenia
ICAM-1	Intercellular adhesion molecule-1
IGFBP-1	Insulin-like growth factor binding protein-1
IL-1β	Interleukin-1 beta
IL-6	Interleukin-6
ISSHP	International Society for the Study of Hypertension in Pregnancy
ISTH	International Society on Thrombosis and Haemostasis
IU	International units
IUGR	Intrauterine growth restriction
LMWH	Low-molecular-weight heparin
MMP	Matrix metalloproteinase
NLR	Neutrophil-to-lymphocyte ratio
OD	Once daily
OR	Odds ratio
PAI-1	Plasminogen activator inhibitor-1
PAPP-A	Pregnancy-associated plasma protein A
PE	Preeclampsia
PI	Pulsatility index
PlGF	Placental growth factor
PLR	Platelet-to-lymphocyte ratio
PP13	Placental protein 13
RCT	Randomized controlled trial
RCOG	Royal College of Obstetricians and Gynaecologists
RR	Relative risk
SB	Stillbirth
sEng	Soluble endoglin
sFlt-1	Soluble fms-like tyrosine kinase-1
SGA	Small for gestational age
SII	Systemic immune inflammation index
sICAM-1	Soluble intercellular adhesion molecule-1
SOGC	Society of Obstetricians and Gynaecologists of Canada
TF	Tissue factor
TFPI	Tissue factor pathway inhibitor
TNF-α	Tumor necrosis factor-alpha
tPA	Tissue plasminogen activator
VCAM-1	Vascular cell adhesion molecule-1
VEGF	Vascular endothelial growth factor
VTE	Venous thromboembolism
